# One Small RNA of *Fusarium graminearum* Targets and Silences *CEBiP* Gene in Common Wheat

**DOI:** 10.3390/microorganisms7100425

**Published:** 2019-10-09

**Authors:** Jiao Jian, Xu Liang

**Affiliations:** 1 Institutes of Science and Development, Chinese Academy of Sciences, Beijing 100190, China; 2 Beijing DaXing District Forestry Workstation, No. 17 Administrative Street, Huangcun Town, Beijing 102600, China; xuliang19824@163.com

**Keywords:** small RNA, wheat, *fusarium graminearum*, gene silencing

## Abstract

The pathogenic fungus *Fusarium graminearum* (*F. graminearum*), causing *Fusarium* head blight (FHB) or scab, is one of the most important cereal killers worldwide, exerting great economic and agronomic losses on global grain production. To repress pathogen invasion, plants have evolved a sophisticated innate immunity system for pathogen recognition and defense activation. Simultaneously, pathogens continue to evolve more effective means of invasion to conquer plant resistance systems. In the process of co-evolution of plants and pathogens, several small RNAs (sRNAs) have been proved in regulating plant immune response and plant-microbial interaction. In this study, we report that a *F. graminearum* sRNA (*Fg-sRNA1*) can suppress wheat defense response by targeting and silencing a resistance-related gene, which codes a Chitin Elicitor Binding Protein (*TaCEBiP*). Transcriptional level evidence indicates that *Fg-sRNA1* can target *TaCEBiP* mRNA and trigger silencing of *TaCEBiP* in vivo, and in *Nicotiana benthamiana* (*N. benthamiana*) plants, Western blotting experiments and YFP Fluorescence observation proofs show that *Fg-sRNA1* can suppress the accumulation of protein coding by *TaCEBiP* gene in vitro. *F. graminearum* PH-1 strain displays a weakening ability to invasion when Barley stripe mosaic virus (BSMV) vector induces effective silencing *Fg-sRNA1* in PH-1 infected wheat plants. Taken together, our results suggest that a small RNA from *F. graminearum* can target and silence the wheat *TaCEBiP* gene to enhance invasion of *F. graminearum*.

## 1. Introduction

At present, widely recognized mechanisms of plant disease resistance are divided into two categories [1,2,3,4]. The first layer employs pattern recognition receptors (PRRs) to detect conserved pathogen-associated molecular patterns (PAMP) and to trigger PAMP-triggered immunity (PTI) [5,6,7,8,9]. The second layer, effector-triggered immunity (ETI), involves a rapid and robust defense activation triggered by the direct or indirect recognition between an isolate-specific pathogen avirulence (Avr) effector and its cognate host resistance (R) protein, often accompanied by a hypersensitive reaction (HR) at the attempted pathogen infection sites, which activates a set of innate immunity signaling pathways [10,11,12,13,14].

Recently, small RNAs (sRNAs) from pathogens have been found to secrete to host plants, causing cross-species of RNA interference reaction and leading to a loss of function of the host plant resistance [15]. The biological function of these small RNA molecules from pathogens is similar to non-toxic effector proteins. For example, some *Botrytis cinerea* sRNAs have been proved to hijack the host RNA interference (RNAi) machinery by loading into *Arabidopsis* Argonaute 1 to selectively silence host immunity genes, demonstrating that a fungal pathogen transfers “virulent” sRNA effector into host cells to achieve infection, which reveals a naturally occurring cross-kingdom RNAi [15]. Additionally, *Arabidopsis* cells also have been proved to secrete exosome-like extracellular vesicles to deliver sRNAs into fungal pathogen *Botrytis cinerea*. Transferred host sRNAs induce silencing of fungal genes critical for pathogenicity. Thus, *Arabidopsis* has adapted exosome-mediated cross-kingdom RNA interference as part of its immune responses during the evolutionary arms race with the pathogen [16]. In addition, wheat microRNA (miRNA) related research shows that, Tae-miR1023 can suppress the invasion of *Fusarium graminearum* (*F. graminearum*) by targeting and silencing *FGSG_03101* which codes an alpha/beta hydrolase gene in *F. graminearum* [17]. However, there are no reports on whether endogenous sRNAs from *F. graminearum* can be transported into common wheat and play a biological role. In terms of the two main types of sRNAs (siRNAs and miRNAs), although they differ in their biosynthetic mechanisms [18], they are extremely similar in terms of product size, sequence characteristics, and specific silencing patterns, which implies that there are inevitable similarities between the biological functions and mechanisms of siRNAs and miRNAs.

The traditional methods of plant disease research techniques commonly used are host-induced gene silencing (HIGS), which is a method of reverse genetics technique widely used. It can be artificially induced by pathogens associated with double stranded RNA fragments, so that plants get new disease-resistant function via HIGS [19,20]. For example, using a gene gun bombardment, transient expression barley powdery mildew toxic effector gene AVRa10 of RNA fragments in barley leaves, can effectively inhibit the barley powdery mildew infection of barley [20]. *Agrobacterium tumefaciens* expression of mitogen activated protein kinase RNA fragments can effectively enhance the wheat leaf rust resistance [21]. By stable transgenic methods, the *F. graminearum* cytochrome P450 lanosterol C-14α-demethylase (CYP51) genes fragment was stably transformed into *Arabidopsis* and barley plants, and found that stable transgenic plants obtained for resistance to *F. graminearum* by means of HIGS [22]. These HIGS technology applications are based on artificially induced plant pathogens which produce exogenous siRNAs, however, a direct over-expression or silencing of *F. graminearum* small RNA molecules in common wheat has not been found [23].

In order to detect whether *F. graminearum* endogenous sRNAs can be transferred into wheat to exert a biological function, we decided to screen *F. graminearum* sRNAs, which could target the wheat genome, and investigated the effect of silencing of target candidate genes. Fortunately, we found one *F. graminearum* endogenous sRNA could target the wheat *CEBiP* gene, and negatively regulate wheat resistance.

## 2. Materials and Methods

### 2.1. Plant Materials

*Nicotiana benthamiana* (*N*. *benthamiana*) plants are grown in a controlled environment at 25 °C with a 14-h-light/10-h-darkness photoperiod. Wheat plants (Chinese spring) used for the BSMV-based sRNA silencing experiment and *F. graminearum* (strain PH-1) punch inoculation experiment are grown in pots in a greenhouse with 16-h-light/8-h-darkness cycle until the two-leaf stage. After inoculated with BSMV, wheat plants are transferred to a climate chamber at 23–25 °C for the evaluation. For each biological replicate, six wheat seeds are sown in one pot of 12 cm diameter, and two pots per BSMV construct. Totally, 10–12 wheat plants of two-leaf stage are prepared for BSMV inoculation. Twenty segments of 4th wheat leaves displaying the BSMV infected symptom, are collected from three biological replicates for the *F. graminearum* punch inoculation experiment.

### 2.2. Small RNA Isolation and Deep Sequencing

Fifteen-day-old leaves of wheat were inoculated with *F. graminearum* strain PH-1 for 0, 24 and 72 h, and total RNAs were isolated using TRIzol solution according to the manufacturer’s instructions. Small RNAs of 18–30 nt were excised and isolated from 5 to 10 mg total RNAs electrophoresed on 15% polyacrylamide denaturing gel, and then were ligated with 59 nt and 39 nt adapters (BGI, Beijing). The ligated small RNAs were used as templates for cDNA synthesis followed by PCR amplification, and synthetic cDNA were prepared for sequencing. The obtained libraries were sequenced using the Solexa sequencing platform (BGI, Beijing).

### 2.3. Fungal Strains, Culture Conditions and Punch Inoculation Experiment

*F. graminearum* strain PH-1 is used as the wild-type (WT) strain in this study. The WT strains are routinely cultured on potato dextrose agar (PDA) (200 g potato, 20 g dextrose, 20 g agar and 1 L water) at 25 °C with a 12-h-light/12-h-darkness cycle. The WT strains are grown on carrot agar for induction of sexual development near-UV light (wavelength, 365 nm; HKiv Co., Ltd., Xiamen, China), and in mung bean broth (MBB) for conidiation assays under continuous light. Assays for *F. graminearum* punch inoculation are performed as described previously [24]. For all leaf inoculation assays, the *F. graminearum* conidia concentration is adjusted to 5 × 10^4^ conidia per mL^−1^. Inoculations of 4th wheat leaves displaying Barley stripe mosaic virus (BSMV) symptom, or non-BSMV infected wheat leaves, are done by wound inoculation of detached leaves segments with *F. graminearum* strains. Fifteen leaves are detached for each biological replicate and transferred in 1% agar plates supplemented with 85 μM Benzimidazole. For assessing the progression of *F. graminearum* disease symptoms, the lesion size is measured from the digital images using the free software ImageJ program (http://rsb.info.nih.gov/ij/index.html). Each experiment is repeated three times.

### 2.4. Vector Constructions

For construct BSMV: OE-FgsRNA1, *Fg-sRNA1* is engineered into *Osa-miR528* precursor backbone using overlap PCR to replace endogenous miRNA sequence. Both miRNA and the partially complementary miRNA^*^ sequences in *Osa-miR528* precursor, are substituted by *Fg-sRNA1* and Fg-sRNA1^*^, respectively. The reconstructed precursor is added ligation-independent-cloning (LIC) adaptors for linking with BSMV vector. For construct BSMV: STTM-FgsRNA1 (for silencing of *Fg-sRNA1* using short tandem target mimic (STTM) strategy) is constructed as follows. Primers with LIC adaptor, corresponding target mimic of *Fg-sRNA1*, and STTM 48 nt spacer (5′-GTTGTTGTTGTTATGGTCTAATTTAAATATGGTCTAAAGAAGAAGAAT-3′) are employed to PCR amplify STTM-FgsRNA1 molecules. STTM-FgsRNA1 is added LIC adaptors for linking with BSMV vector. The *Osa-miR528* precursor harboring *Fg-sRNA1* fragment or STTM fragment with LIC adaptors are cloned into BSMV-γb using the LIC protocol as described [25]. The plasmids in Western blotting experiment and YFP observation assay are constructed by Gateway technology (Invitrogen Thermo Fisher Scientific-CN) following the instructions of the manufacturer (http://www.invitrogen.com/content/sfs/manuals/gatewayman.pdf). The two 35S: pKANNIBAL-FgsRNA1 or -FgsRNA2 expressing vectors are constructed by PCR amplification of *Osa-miR528* precursor harboring *Fg-sRNA1*, or *Fg-sRNA2*, followed by sequential digestion with *Hind*III and *Kpn*I and subsequent cloning into the pKANNIBAL destination vector. All constructs are confirmed by DNA sequencing.

### 2.5. BSMV-Based Experiments

BSMV-based sRNA over-expression and silencing experiments are performed as described [17,25]. Constructs of pCaBS-α, pCaBS-β, and pCaBS-γ-LIC derivatives (OE-FgsRNA1 and STTM-FgsRNA1) are transformed into *Agrobacterium* (*A. tumefaciens* strain EHA105). The *Agrobacterium* suspensions of OD_600_ = 0.8 are mixed at 1:1:1 ratio (pCaBS-α: pCaBS-β: each pCaBS-γ-LIC derivative) and infiltrated in *N. benthamiana* leaves. Agroinfiltrated *N. benthamiana* leaves can provide excellent sources of virus for secondary BSMV infections in wheat plants. The *N. benthamiana* sap is extracted from leaves with BSMV symptom at about 12 days post infiltration, ground in 20 mM Na-phosphate buffer (pH 7.2) containing 1% celite, and the sap is mechanically inoculated onto the first two emerging leaves of wheat. Infected wheat plants are further grown for 14–21 d to allow emergence of new leaves displaying viral symptoms. Segments of the 4th leaves of BSMV-infected wheat plants are collected for further experiments from three biological replicates per construct.

### 2.6. Protein Analyses

Western blotting experiment is performed in order to analyze whether *Fg-sRNA1* or *Fg-sRNA2* can cleavage TaCEBiP, respectively. First, *35S*: pKANNIBAL-FgsRNA1 or -FgsRNA2 expressing vectors are transiently transformed into *N. benthamiana* leaves by *Agrobacterium*-mediated transformation. After 24 hpi, *35S*: cTAPi-TaCEBiP or -TaCEBiP mutant (TaCEBiP-m) proteins expressing vector are agroinfiltrated into the same *N. benthamiana* leaves, respectively. For TaCEBiP & TaCEBiP-m proteins accumulation analysis, leaf samples are collected at 24–36 h post the 2nd agroinoculation. HA-tagged protein extraction, separation and fraction are detected by immunoblotting using rat anti-HA antibody (Roche, Indianapolis, IN, USA) and anti-rat IgG conjugated with horseradish peroxidase (HRP) (Sigma, St Louis, MO, USA). For YFP observation assay, cell suspensions of *A. tumefaciens* strain GV3101 containing the indicated constructs were infiltrated into *N. benthamiana* leaves. Confocal images were taken using a confocal laser-scanning microscope Zeiss LSM 710 (Carl Zeiss, Oberkochen, Germany).

### 2.7. RNA, DNA and PCR Analysis

Plant total RNAs are extracted from three independent biological replicates, BSMV-infected leaves and *F. graminearum*-infected lesion area of wheat leaves with TRIzol reagent, as described by the manufacturer (Invitrogen Thermo Fisher Scientific-CN, Shanghai, China), and treated with DnaseI. DNA and Total RNA are extracted from cultured *F. graminearum* strains using fungal DNA or Total RNA extraction kits. About 2 mg of total RNA and M-MLV Reverse Transcriptase (Promega) are further used for reverse transcription. For coding gene (*TaCEBiP*) reverse transcription, first-strand cDNA is synthesized using Oligo (dT)_18_. For sRNA reverse transcription, specifically designed end-point stem-loop reverse transcription primers are used, and follow the procedures described by Liu [26]. Real-time RT-PCR assays with three technical replicates are performed using StepOne real-time system (Applied Biosystems, Foster City, CA, USA) and GoTaq qPCR Master Mix (Promega, A6001, Madison, WI, USA). sRNA forward primers are respectively used with universal reverse primer to quantify the relative transcript levels of mature *Fg-sRNA1* or *Fg-sRNA2*. Real-time RT-PCR components for sRNA are as follows: 2× GoTaq qPCR Master Mix 5 μL, diluted cDNA 1 μL, sRNA forward primer 0.2 μL, sRNA universal reverse primer 0.2 μL, and ddH_2_O up to 10 μL. Real-time RT-PCR conditions are as follows: 95 °C for 5 min, followed by 35–40 cycles of 95 °C for 5 s, 60 °C for 10 s, and 72 °C for 1 s. For melting curve analysis, denature samples at 95 °C, then cool to 65 °C at 20 °C per second [17,26]. For the determination of target gene *TaCEBiP*, gene-specific primer pairs spanning the miRNA-guided cleavage site are used. *Tae-U6*, *Tae-Actin* and *Fg-Actin* which served as internal reference genes for sRNAs and protein-coding genes are detected, respectively. GenBank accession numbers of *Tae-U6*, *Tae-Actin*, *Fg-Actin* are X63066, KC775781, XM_011328784.1, respectively. Error bars representing standard error (SE) are calculated from three biological replicates per construct.

## 3. Results

### 3.1. Construction of Fusarium graminearum sRNA Library and Alignment with Wheat Genome

To explore the role of *F. graminearum* sRNAs in regulation of host–pathogen interaction, we profiled the sRNA library prepared from *F. graminearum* (strain PH-1) total biomass after three days of culture (using potato dextrose agar). sRNA libraries prepared from *F. graminearum* infected wheat leaves, collected at 0, 24 and 72 h after inoculation, were used as controls. A total of 12.7 million raw reads ranging in size from 18 to 30 nucleotide (nt) were generated. We focused on sRNAs with more than 100 reads per million sRNA reads in sRNA libraries. After removing adaptor sequences, we used BLAST of sRNAs against the Rfam database to remove noncoding RNAs such as rRNA, tRNA, snRNA and snoRNA. A total of 4139 potential sRNAs were obtained by further bioinformatics analysis, with sequences exactly matching the *F. graminearum* genome (NCBI reference sequence: NC_026474.1) [27]. We then mapped these sRNA candidates to the wheat reference genome (GenBank assembly: GCA_900519105.1) [28] and identified 264 sRNAs from *F. graminearum* sRNA library target wheat genome with 0–3 nucleotide mismatch, compared with the control sRNAs libraries.

**Figure 1:**
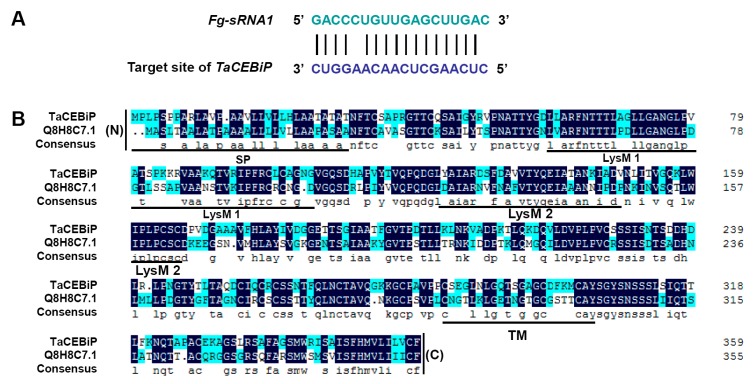
Sequence information of *Fg-sRNA1* and *TaCEBiP*. (**A**) Schematic diagram of *Fg-sRNA1* (green color) and its target site (blue color) in *TaCEBiP* of common wheat; and (**B**) alignment of the amino acid sequences between wheat *TaCEBiP* and Q8H8C7.1 (rice *OsCEBiP*). The number on the right represents the number of amino acids. Putative coding sequence of *TaCEBiP* was aligned with rice Q8H8C7.1. Identical amino acids are highlighted with black boxes. SP = signal peptide; LysM 1/LysM 2, LysM motif; TM = transmembrane region; (N) = N-terminus; and (C) = C-terminus.

Among them, one 18 nt length sRNA (*Fg-sRNA1*) from *F. graminearum* target a wheat endogenous gene, as seen in Figure 1A, coding a CEBiP (Chitin Elicitor Binding Protein), which is likely to function in a wheat disease resistance signaling pathway [29]. Chitin is a major structural component of fungal cell walls and is therefore likely to function as a PAMP [29]. By homologous cloning, we obtained the *CEBiP* gene coding sequence. Analysis by amino acid sequence alignment showed that the predicted amino acid sequence of the CEBiP displayed a high identity to rice CEBiP (Q8H8C7.1). Furthermore, this sequence contained a signal peptide at the N-terminus, two LysM motifs, and a transmembrane region in the C-terminal region, which are all present in rice CEBiP. Therefore, we consider this gene is very likely to be orthologous to rice *CEBiP*, and accordingly designated the gene *TaCEBiP*, as shown in Figure 1B.

**Figure 2:**
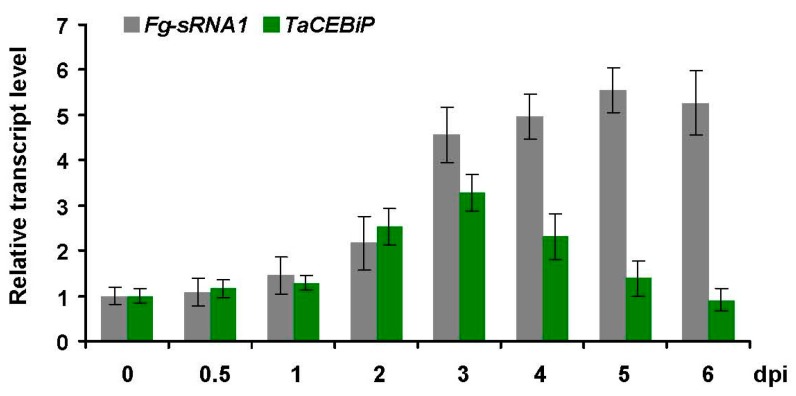
Expression profiling of *Fg-sRNA1* and *TaCEBiP*. Detection of relative transcript level of *Fg-sRNA1* and *TaCEBiP* from *F. graminearum* infected lesion area in wheat leaves by real-time quantitative PCR (RT-qPCR) assay. At 0, 0.5, 1, 2, 3, 4, 5 and 6 days post inoculation (dpi) of *F. graminearum*, wheat leaves were collected for RT-qPCR detection.

To test whether *F. graminearum* sRNAs *Fg-sRNA1* could indeed suppress wheat genes during infection, *Fg-sRNA1* was further characterized. We conducted real-time quantitative PCR (RT-qPCR) analysis to detect the relative transcript level of *Fg-sRNA1* and *TaCEBiP* during *F. graminearum* infection, and found *Fg-sRNA1* was enriched after *F. graminearum* infection, and expression of *TaCEBiP* increased gradually until three days post inoculation (dpi) and then decreased, as shown in Figure 2. This result showed that *Fg-sRNA1* and *TaCEBiP* were involved in the process of *F. graminearum* infection.

### 3.2. Fg-sRNA1 Triggers Silencing of TaCEBiP that is Involved in F. graminearum Pathogenicity

The technology about host-induced gene silencing (HIGS) targeting fungal genes has been developed in several plant-microbial interactions [20]. In addition, Barley stripe mosaic virus (BSMV) has a tripartite genome, composed of α, β, and γ RNAs [21]. BSMV has become a popular vector for virus-induced gene silencing (VIGS), virus-mediated over-expression of heterologous protein (VOX), and HIGS in barley and wheat. In wheat, BSMV vectors have been used to assess the possibility of controlling devastating *Fusarium* diseases via HIGS of the fungal *CYP51* genes, and demonstrated that silencing of an azole fungicide target was highly efficient in controlling fungal growth [22]. To determine whether *F. graminearum* sRNAs *Fg-sRNA1* could trigger silencing of wheat endogenous gene *TaCEBiP*, we examined the transcript level of *TaCEBiP* after BSMV-induced *Fg-sRNA1* over-expression. A previous study found that BSMV expressing Tae-miR159a precursor could produce virus small interfering RNA (vsiRNA) from the same miRNAs generating sites of Tae-miR159a precursor and down regulate its target gene *TaMYB3* in vivo [30].

**Figure 3:**
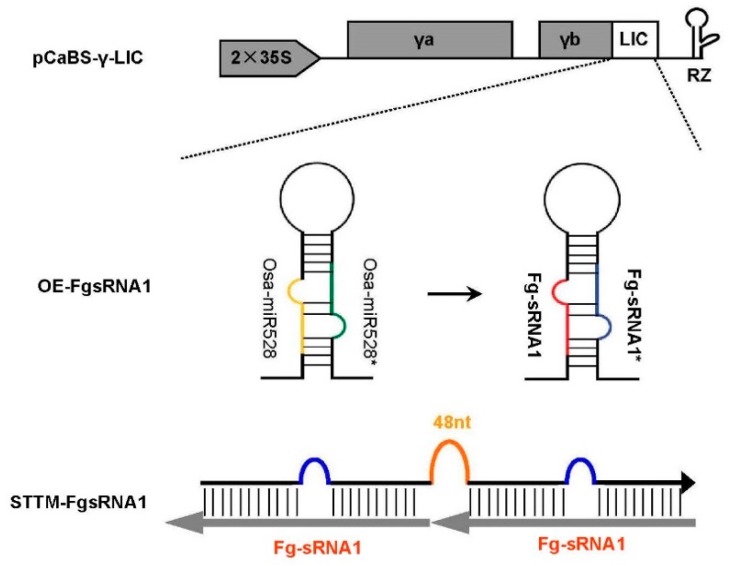
Schematic diagram of integrating BSMV-γb vector and *Osa-miR528* precursor harboring *Fg-sRNA1* or STTM sequences. Modified BSMV-γb vector (pCaBS-γ-LIC) was shown in this figure. *Osa-miR528* precursor harboring *Fg-sRNA1* or STTM sequences can be cloned into pCaBS-γ-LIC derivatives by the LIC reaction. OE-FgsRNA1 structure contained an *Osa-miR528* precursor backbone, but *Osa-miR528* and Osa-miR528* were changed to *Fg-sRNA1* and Fg-sRNA1^*^. STTM-FgsRNA1 structure contained two tandem target mimics separated by a 48 nt imperfect stem-loop linker.

**Figure 4:**
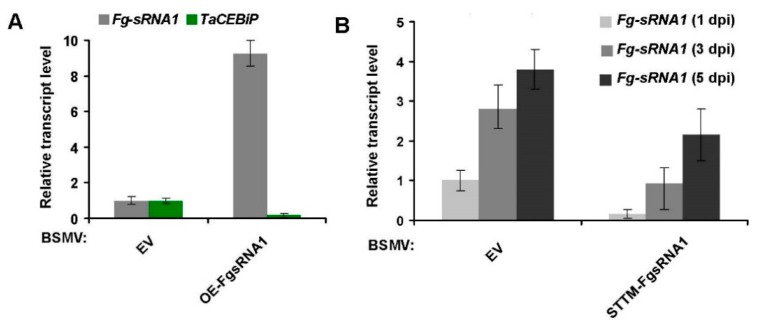
Detection of relative transcript level of *Fg-sRNA1* and *TaCEBiP* from *F. graminearum* infected lesion area in BSMV pre-inoculated wheat leaves. (**A**) BSMV-based experiment procedures can be found in Materials and Methods section. After 15th day post BSMV constructs inoculation of wheat plants, segments of the 4th leaves of BSMV-infected (BSMV: EV&BSMV: OE-FgsRNA1) wheat plants were collected and then inoculated with *F. graminearum* (strain PH-1) by the punch inoculation method. Relative transcript levels of *Fg-sRNA1* and *TaCEBiP* were detected by stem-loop RT-PCR and real-time qPCR assay at 1 dpi. Error bars represented standard error (SE) of three representing experiments from four replicates; and (**B**) similar to BSMV: OE-FgsRNA1infection and punch inoculation assay, BSMV-infected (BSMV: EV&BSMV: STTM-FgsRNA1) wheat leaves were collected and then inoculated with *F. graminearum* by the punch inoculation method. Relative transcript levels of *Fg-sRNA1* were detected by stem-loop RT-PCR at 1, 3, 5 dpi, respectively. Error bars represented standard error (SE) of three representing experiments from four replicates.

**Figure 5:**
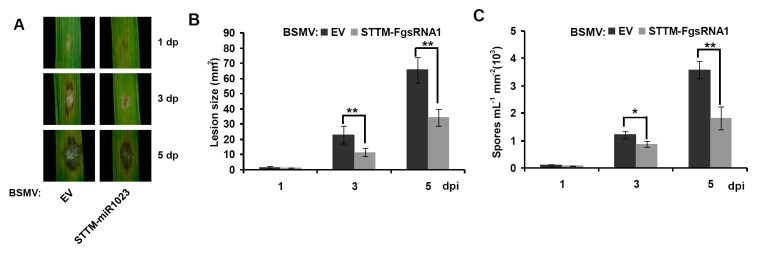
Analysis of lesion size of wheat leaves and *F. graminearum* spores number after BSMV induced *Fg-sRNA1* silencing. (**A**) Lesion area phenotypes of *F. graminearum* infected wheat leaves which were pre-inoculated by BSMV empty vector (BSMV: EV) and BSMV: STTM-FgsRNA1, were photographed at 1, 3 and 5 dpi. BSMV = EV infected wheat plants were used as controls; (**B**) lesion-size (mm^2^) of corresponding different treatments; and (**C**) number of *F. graminearum* spores produced by lesions on corresponding treated wheat leaves. Error bars representing SE were calculated from three replicates. Significance was determined at * *p* < 0.05 and ** *p* < 0.01 (*n* ≥ 3) with a *t*-test.

Here, we used the modified BSMV vector to express *Osa-miR528* precursor, and short tandem target mimic (STTM) against *Fg-sRNA1*, and then cloned it into pCaBS-γ-LIC vector to generate BSMV: OE-FgsRNA1 and BSMV: STTM-FgsRNA1 constructs, respectively, as seen in Figure 3. BSMV-based experiment procedures can be found in the Materials and Methods section. After the 15th day post BSMV constructs inoculation of wheat plants, segments of the 4th leaves of BSMV-infected wheat plants were collected and inoculated with *F. graminearum* (strain PH-1) by the punch inoculation method. Relative transcript levels of *Fg-sRNA1* and *TaCEBiP* were detected. Lesion size of wheat leaves and *F. graminearum* spores number were counted at 1, 3 and 5 dpi (punch inoculation). Stem-loop RT-PCR together with real-time PCR assays showed an increased relative transcript level of *Fg-sRNA1* and a decline expression of *TaCEBiP* in BSMV: OE-FgsRNA1 infected plants, as seen in Figure 4A, and a down-regulated relative transcript level of *Fg-sRNA1* in BSMV: STTM-FgsRNA1 infected plants, at 1, 3 and 5 dpi (punch inoculation), as seen in Figure 4B. BSMV empty vector (BSMV: EV) infected wheat plants were used as controls. Lesion size of wheat leaves and *F. graminearum* spores number were significantly lower in BSMV: STTM-FgsRNA1 infected plants, at 1, 3 and 5 dpi (punch inoculation), respectively, as seen in Figure 5. These results indicate that BSMV-based sRNA technology can effectively over-express or silence *Fg-sRNA1* in vivo, and *Fg-sRNA1* does significantly affect the transcriptional expression of *TaCEBiP* gene.

### 3.3. Fg-sRNA1 Affects Accumulation of Protein Encoded by TaCEBiP Gene In Vivo

**Figure 6:**
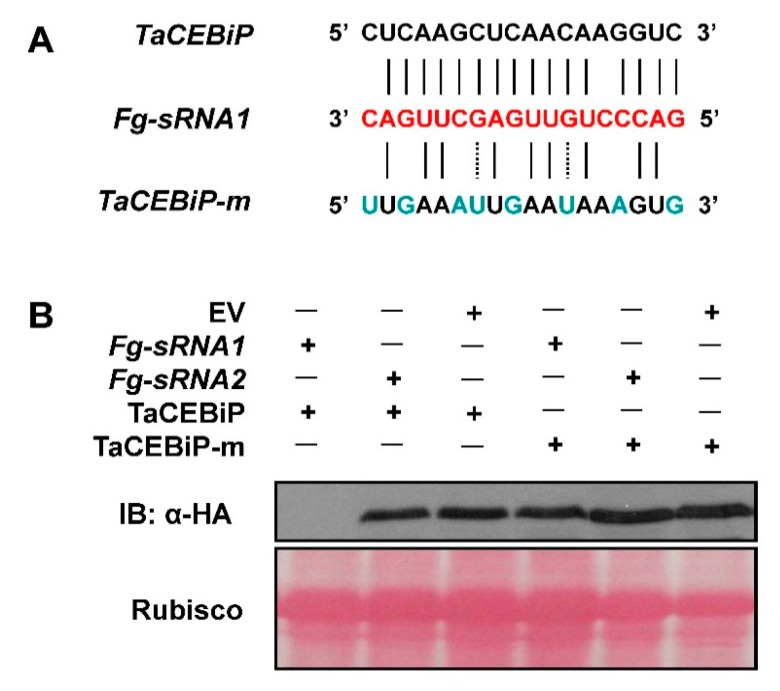
*Fg-sRNA1* affects accumulation of TaCEBiP in vivo. (**A**) Target site and target site synonymously mutated versions of *TaCEBiP* were used in this study; and (**B**) in *N. benthamiana*, co-expression of *Fg-sRNA1* with its targets (HA-tagged), TaCEBiP or TaCEBiP-m, revealed target silencing by means of Western blot analysis. Co-expression of *Fg-sRNA1* with different versions of targets were used as controls.

To further confirm that the suppression of *TaCEBiP* was indeed triggered by *Fg-sRNA1*, we performed co-expression assays in *N*. *benthamiana*. Another sRNA (Sequence information: 5′ UGCAGAUCUUGGUGGUAGUAG3′) from *Fusarium graminearum* sRNA library was selected as a control. There is no reverse complementation between this sRNA (*Fg-sRNA2*) and *TaCEBiP* mRNA chain, although *Fg-sRNA2* can target other locations in wheat genome, based on our sequence alignment analysis. Expression of hemagglutinin (HA)–epitope tagged TaCEBiP was reduced when they were co-expressed with the corresponding *Fg-sRNA1* but not when co-expressed with *Fg-sRNA2*, which shared no sequence similarity, as shown in Figure 6B. The silencing was abolished, however, when the target gene *TaCEBiP* carried a synonymously mutated version of the relevant *Fg-sRNA1* target sites, as seen in Figure 6A,B.

**Figure 7:**
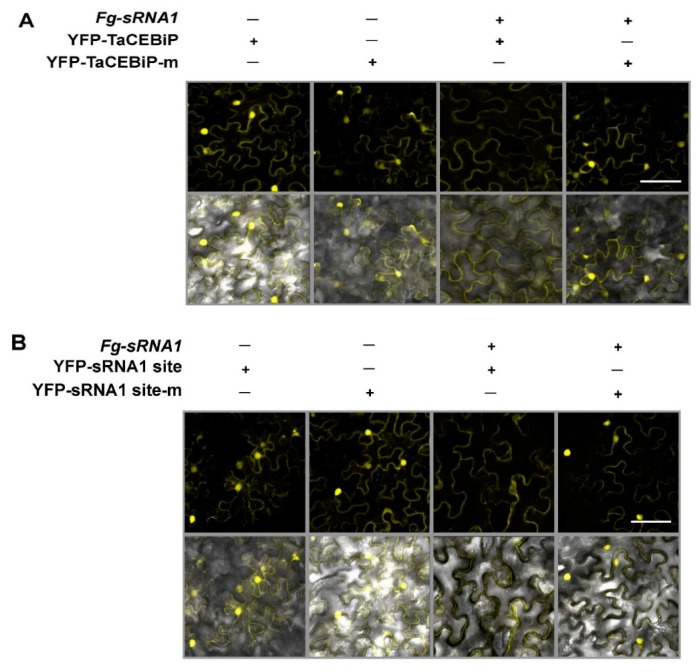
Suppression of YFP–tagged *TaCEBiP* co-expressed with *Fg-sRNA1*. (**A**) Co-expression of YFP-TaCEBiP or its synonymously mutated version (YFP-TaCEBiP-m) with *Fg-sRNA1* were observed with confocal microscopy; and (**B**) Expression of the YFP sensors carrying a *Fg-sRNA1* target site of TaCEBiP or a *Fg-sRNA1* target site-m, were analyzed after co-expression of *Fg-sRNA1*. Samples were examined at 48 h after transiently *Agrobacterium*-mediated transformation into *N. benthamiana* leaves. (**Top**) YFP. (**Bottom**) YFP/bright field overlay. Scale bar is 50 μm. Similar results were obtained in three biological replicates.

We also observed suppression of yellow fluorescent protein (YFP)–tagged TaCEBiP co-expressed with *Fg-sRNA1*. When the *Fg-sRNA1* target sites of *TaCEBiP* was mutated, co-expression of *Fg-sRNA1* failed to suppress expression of YFP fusion protein, as shown in Figure 7A. Similarly, of the YFP-sensors with wild-type or mutated *Fg-sRNA1* target sites, only the wild-type sensor was suppressed after co-expression of *Fg-sRNA1,* as shown in Figure 7B. Thus, *Fg-sRNA1* indeed triggers silencing of *TaCEBiP* and affects accumulation of TaCEBiP.

## 4. Discussion

Our research showed that a small RNA *Fg-sRNA1* of *F. graminearum* can cross-species transport and cause silence of wheat resistance-related gene *TaCEBiP*. BSMV-mediated *Fg-sRNA1* over-expression and silencing systems have been employed in this study. For example, enhanced expression of *Fg-sRNA1* results in a larger area of wheat leaf necrosis phenotype, and pre-expression of STTM molecules that silence *Fg-sRNA1* can effectively reduce the transcriptional expression of *Fg-sRNA1* after fungal infection, and effectively prevent the growth of necrotic spots in wheat leaves. Relative transcriptional level detection experiments indicate that *Fg-sRNA1* can target *TaCEBiP* mRNA and trigger silencing of *TaCEBiP* in vivo, and Western blotting experiments and YFP fluorescence observation proofs show that *Fg-sRNA1* can suppress the accumulation of TaCEBiP. Overall, our results suggest that *Fg-sRNA1* of *F. graminearum* can target and silence wheat resistance-related gene *TaCEBiP* to enhance invasion of *F. graminearum* accompanying with the weakening of wheat resistance. Combined with previous reports that wheat miRNA1023 can be transported to *F. graminearum* and fungal infection by silencing *F. graminearum* endogenous gene [12], our research indicates that some sRNAs are present in the trans-species transport and regulation mechanisms between common wheat and *F. graminearum*.

The main mechanism of plant disease resistance PTI and ETI can be grouped into four stages of immune response [1,2]. The first stage, the plant transmembrane pattern recognition receptors (Pattern recognition receptors, PRRs) recognize pathogen associated molecular model (Pathogen-associated molecular patterns, PAMPs), such as bacterial flagellin, and lead to pathogen associated molecular models of induced immune response to inhibit further colonization and spread of pathogens [2,4]. In stage two, successful pathogens deploy effectors that contribute to pathogen virulence. Effectors can interfere with PTI. This results in effector-triggered susceptibility (ETS) [3,5]. In the third stage, the plant will not sit still, evolved more antiviral protein, directly or indirectly identify the pathogen effector proteins, and trigger effector proteins induced immune response (Effector-triggered immunity, ETI). ETI is an accelerated and amplified PTI response, resulting in disease resistance and, usually, a hypersensitive cell death response (HR) at the infection site [7,10,11]. The fourth stage, co-evolution of plants and pathogens, namely natural selection process, are other types of pathogenic bacteria by secreting effector protein or modifying the original effector proteins to break through the plant’s defense system, and the plant also evolved new antiviral proteins responding to new pathogen effector proteins, forward with this process of repeated co-evolution [12].

Animal and plant pathogens have evolved virulence or effector proteins to counteract host immune responses. Various protein effectors have been predicted or discovered in fungal or oomycete pathogens from whole-genome sequencing and secretome analysis, although delivery mechanisms are still under active investigation [12,13,14]. Jin’s lab shows that sRNAs as well can act as effectors through a mechanism that silences host genes in order to debilitate plant immunity and achieve infection. They find that sRNAs from *B. cinerea* hijack the plant RNAi machinery by binding to AGO proteins, which in turn direct host gene silencing. The implications of these findings may extend beyond plant gray mold disease caused by *B. cinerea*, and suggest an extra mechanism underlying pathogenesis promoted by sophisticated pathogens with the capability to generate and deliver small regulatory RNAs into hosts to suppress host immunity [15].

From plant to pathogen small molecule RNA interference effect exercised across species has been reported [21]. For example, using a gene gun bombardment, transient expression barley powdery mildew toxic effector gene AVRa10 of RNA fragments in barley leaves, can effectively inhibit the barley powdery mildew infection of barley [20]. *Agrobacterium tumefaciens* expression of mitogen activated protein kinase RNA fragments, can effectively enhance the wheat leaf rust resistance [21]. By stable transgenic methods, the *F. graminearum* cytochrome P450 lanosterol C-14α-demethylase (*CYP51*) genes fragment was stably transformed into *Arabidopsis* and barley plants, and found that stable transgenic plants obtained for resistance to *F. graminearum* by means of HIGS [22]. These HIGS technology applications were based on artificially induced plant pathogens which produce exogenous siRNAs. Compared with these studies, a direct cross-kingdom expression of fungal endogenous small RNA molecule and acquired enhanced infectivity were reported in this study.

BSMV-based sRNA silencing and over-expression technology is the most widely used reverse-genetic strategy to study sRNA function [31]. Transient virus-induced gene silencing displays several advantages when constitutive loss of gene function through stable transformation brings about sporophytic or gametophytic lethality [31]. Moreover, the currently described BSMV-mediated sRNA silencing and over-expression system is efficient and quick, and it can be carried out for sRNAs silencing through intermediary of argoinocubated *N. benthamiana*, a more simple but effective method without complicated experiment operations or expensive instruments [25]. Here, we presented that BSMV-based sRNA silencing and over-expression system could be used to evaluate the function of *F. graminearum* sRNA by simple agroinfiltration. The modified BSMV vector may facilitate to high-throughput screen the targets of fungal sRNAs, and to characterize their function in wheat crops [25].

This paper verifies the *F. graminearum* small RNA molecule *Fg-sRNA1* capable of transporting into common wheat to exercise RNA interference biological function. A variety of evidences show that *Fg-sRNA1* can target and silence *TaCEBiP* gene, thereby negatively regulating wheat resistance and enhancing *F. graminearum* infection. In summary, this study demonstrates another evidence of co-evolution between *F. graminearum* and main food crops.
